# Growth Curves of Preschool Children in the Northeast of Iran: A Population Based Study Using Quantile Regression Approach

**DOI:** 10.5539/gjhs.v5n3p9

**Published:** 2013-01-15

**Authors:** Abolfazl Payande, Hamed Tabesh, Mohammad Taghi Shakeri, Azadeh Saki, Mohammad Safarian

**Affiliations:** 1 Department of Biostatistics, School of Paramedical Sciences, Shahid Beheshti University of Medical Sciences, Tehran, Iran; 2 Department of Biostatistics and Epidemiology, School of Public Health, Ahvaz Jundishapur University of Medical Sciences, Ahvaz, Iran; 3 Department of Community Medicine, Medical School, Mashhad University of Medical Sciences, Mashhad, Iran; 4 Department of Nutrition, Medical School, Mashhad University of Medical Sciences, Mashhad, Iran

**Keywords:** nonparametric quantile regression, kernel estimation, growth curves, reference curve, child

## Abstract

**Introduction:**

Growth charts are widely used to assess children’s growth status and can provide a trajectory of growth during early important months of life. The objectives of this study are going to construct growth charts and normal values of weight-for-age for children aged 0 to 5 years using a powerful and applicable methodology. The results compare with the World Health Organization (WHO) references and semi-parametric *LMS* method of Cole and Green.

**Methods:**

A total of 70737 apparently healthy boys and girls aged 0 to 5 years were recruited in July 2004 for 20 days from those attending community clinics for routine health checks as a part of a national survey. Anthropometric measurements were done by trained health staff using WHO methodology. The nonparametric quantile regression method obtained by local constant kernel estimation of conditional quantiles curves using for estimation of curves and normal values.

**Results:**

The weight-for-age growth curves for boys and girls aged from 0 to 5 years were derived utilizing a population of children living in the northeast of Iran. The results were similar to the ones obtained by the semi-parametric *LMS* method in the same data. Among all age groups from 0 to 5 years, the median values of children’s weight living in the northeast of Iran were lower than the corresponding values in WHO reference data. The weight curves of boys were higher than those of girls in all age groups.

**Conclusion:**

The differences between growth patterns of children living in the northeast of Iran versus international ones necessitate using local and regional growth charts. International normal values may not properly recognize the populations at risk for growth problems in Iranian children. Quantile regression (QR) as a flexible method which doesn’t require restricted assumptions, proposed for estimation reference curves and normal values.

## 1. Introduction

Reference curves and normal values are often required in medicine (Gannoun, 2002). These curves and values are used for assessment of the general nutritional status of populations of children in diverse settings, as an ancillary tool to screen children for health and nutrition disorders. Also they are a basis for educational materials that promote improved child care by families (Onis, Garza, & Habicht, 1997).

The WHO constructed child growth curves (2003) based on the Box-Cox-Power-exponential (BCPE) method, with curve smoothing by cubic splines for international purposes (Emdadifar, 2008). The application of these curves for international purposes has been challenged due to serious drawbacks on the origin and type of the data set and in the analytical methods of derivations (Abtahi, Doustmohammadian, & Abbdollahi, 2011). There are several methods for obtaining these curves but the simple linear, systematic and efficient statistical methods for constructing them are lacking and also these methods are not robust to outliers (Chen, 2005; Koenker & Hallock, 2001). Quantile regression is an alternative way to create growth curves as an applicable and powerful methodology for estimation of reference curves and normal values of weight-for-age in children, which is an essential component of the children toolkit (Saki, Eshraghian, Mohammad, Foroushani, & Bordbar, 2010; Onis, 2006; Yu & Jones, 1998). So the goal of this study is to construct the growth curves and normal values for children aged 0-5 year old living in Khorasan province, northeast of Iran.

In this study, the non-parametric quantile regression method obtained by local constant kernel estimation of conditional quantiles curves. This method allows quantiles to be estimated as a smooth function of covariates without imposing parametric distributional assumption (Li, Graubard, & Korn, 2010). This further promotes the quantile regression method for constructing growth curves of weight. This non-parametric method can be used as an alternative to parametric and semi-parametric approaches for reference curve estimation. As the proposed approach is robust, the curves and intervals are obtained without outlier detection (Yu, Lu, & Stander, 2003).

## 2. Materials and Methods

The data set of this study consists of cross-sectional measurements on weight for 70737 children aged 0-5 years living in Khorasan province, northeast of Iran. These children were recruited in July 2004 for 20 days from those attending community clinics for routine health checks as a part of a national survey. The selected sample was 11% of the study population. Anthropometric measurements were made by trained health staff using WHO methodology (Linton & Nielsen, 1995).

The non-parametric quantile regression method obtained by local constant kernel estimation of conditional quantiles curves using for estimating of curves and reference values. Quantile regression method was proposed by Koenker and Bassett (1978). The purpose of the quantile regression is to estimate conditional quantile functions, where quantiles of a response variable’s distribution are specified as functions of observed covariates minimizes the weighted sum of the absolute deviations of the error term, unlike ordinary regression models that minimize the sum of the squared residuals. Quantile regression can be parametric or nonparametric. In general, the parametric type is called quantile regression (Koenker, 2005).

In parametric type, when covariates X are considered, the linear conditional quantile function, *Q(τ|X = x) = x′ β(τ)*, can be estimated by solving,


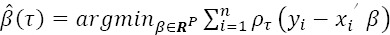


for any quantile *τ ∈ (0,1)*. The quantity β̂(*τ*) is called the *τ*th regression quantile (Buhai, 2005; Wu & Liu, 2009). The non-parametric quantile regression method has the following advantages over semi-parametric LMS method:

(1) There is no distribution assumption.
(2) It is robust in response to outliers.
(3) This method can be applicable to all clinical (or more generally biological) variables that are measured on a continuous scale.

In the present paper, the nonparametric quantile regression method is obtained by local constant kernel estimation of conditional quantiles curves using for estimating of growth curves and normal values. More information of this method is described in greater details in Reference (Koenker, 2005). The proposed regression is estimated using package QUANTREG in the R. Subjective choice method is used for assessing smoothing parameter and Gaussian kernel function is needed during analysis (Jamshidi, Fallah, & Keshavarz, 2005).

## 3. Results

**Figure 1:**
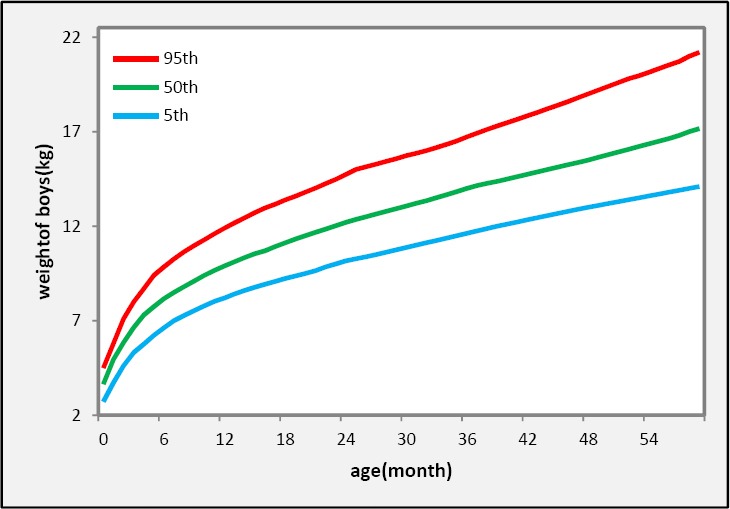
Reference curves in 5^th^, 50^th^ (median) and 95^th^ percentiles obtained with the non-parametric quantile regression (NQR) method for northeast of Iranian boys aged 0 – 5 years

**Figure 2:**
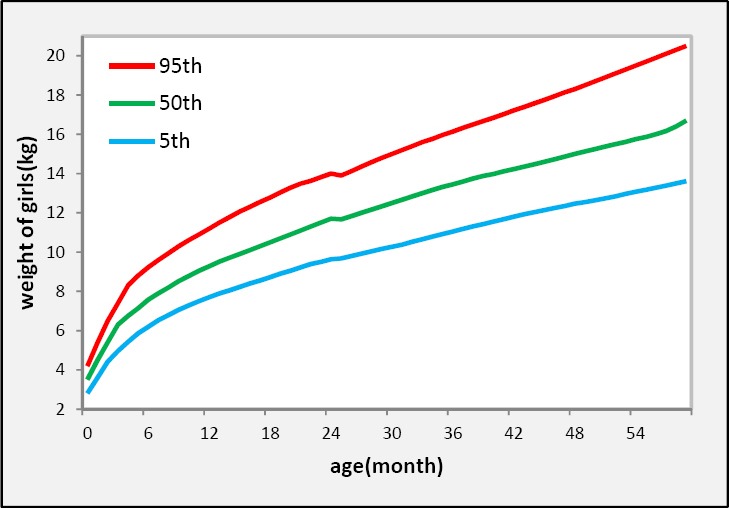
Reference curves in 5^th^, 50^th^ (median) and 95^th^ percentiles obtained with the non-parametric quantile regression (NQR) method for northeast of Iranian girls aged 0 – 5 years

In our data set of 70737 individuals, 36034 (50.9%) are boys and 34703 (49.1%) are girls. Non-normality distribution of weight st each age for both sex groups (P < 0.000) and existing some outliers in the data set, suggesting the proposed and flexible method (quantile regression) to estimate growth curves and reference values of weight for age. Separately, plotting scatter diagrams of weight versus age for boys and girls did not propose any specified pattern. Therefore, used the nonparametric type of quantile regression (NQR) (Wasserman, 2006; Gannoun et al., 2002) supports that boys and girls have different growth patterns, so we construct growth curves separately by sex. Three quantile (5^th^, 50^th^ and 95^th^) curves of weight for boys and girls are shown by figure 1 and 2, respectively.

**Figure 3:**
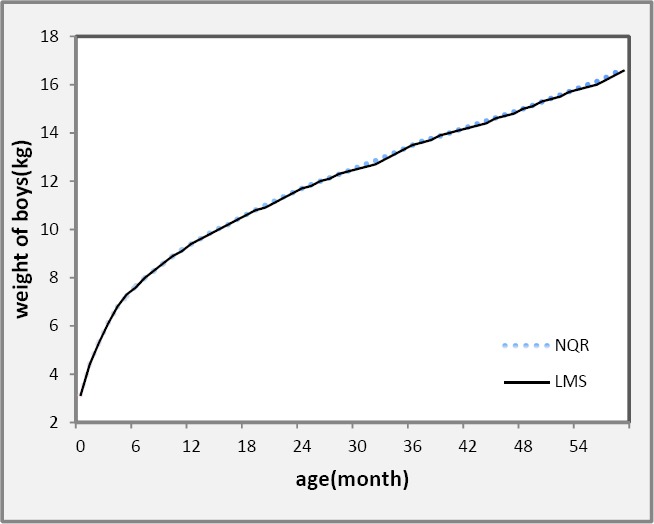
Comparison of semi-parametric LMS and non-parametric quantile regression (NQR) method for 50^th^ percentile (median) of weight using northeast of Iranian boys from birth to 5 years

To provide a visual comparison of the semi-parametric *LMS* method of Cole and Green and NQR methods for estimating growth curves we present in Figure 3 the results for estimated 50 per cent growth curves using two methods for weight.

**Figure 4:**
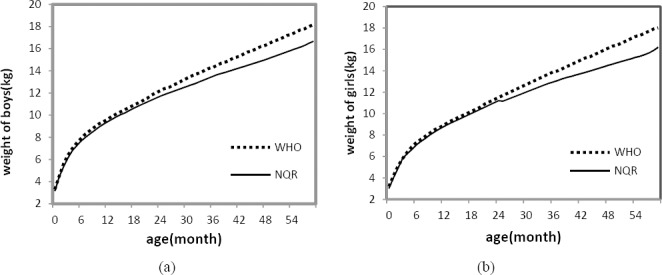
Comparison of WHO growth curves and non-parametric quantile regression (NQR) method for 50^th^ percentile (median) of northeast of Iranian children from birth to 5 years: (a) for boys; (b) for girls

As shown in Figure 3, the results are similar to the ones obtained by the semi-parametric *LMS* method. In this way, this statistical analysis can be of great use for the automatic determination of reference intervals from limited or possibly unreliable data (Onis, 2006). So we compared 50^th^ percentile curves of weight using NQR in the present data set with those obtained by WHO as reference growth curves for boys and girls, separately (see Figure 4). There is an agreement between the two curves from birth to 2 year-old but some differences after 3 year-old increasingly up to 5 in both sexes.

**Figure 5:**
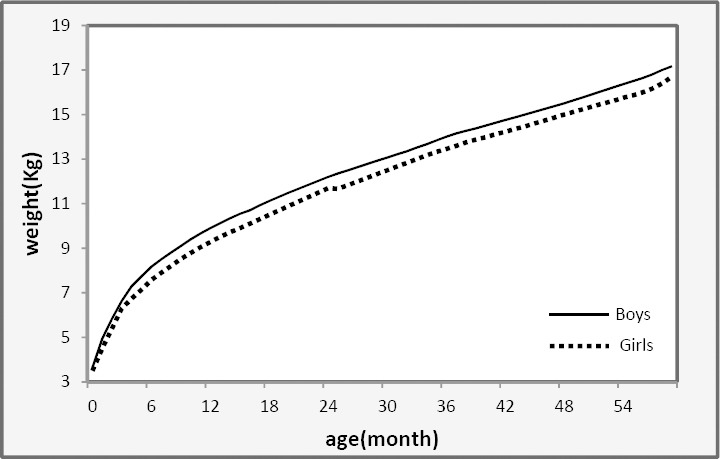
Comparison of boys and girls percentile 50^th^ (median) growth curves of weight using non-parametric quantile regression (NQR) of northeast of Iranian children from birth to 5 years

In order to show the difference between the two growth patterns of boys and girls, comparison of the 50^th^ percentile growth curves estimations of weight might be of great interest. Using the proposed method (NQR) in figure 5 showed that boys growth curves estimations is higher than those for girls in all ages.

For each quantile, the regression quantiles computed at each observed age. The results are shown in Table 1.

**Table 1:** 50th percentiles (median) values of weight(kg)-for-age(mo.) by both non-parametric quantile regression (NQR) estimation method and WHO standard values for boys and girls

	Boys		Girls			Boys		Girls	

Age	Northeast of Iran	WHO	Northeast of Iran	WHO	Age	Northeast of Iran	WHO	Northeast of Iran	WHO
0	3.14	3.3	3.00	3.2	30	12.57	13.3	12.00	12.7
1	4.44	4.5	4.00	4.2	31	12.71	13.5	12.17	12.9
2	5.34	5.6	4.90	5.1	32	12.85	13.7	12.33	13.1
3	6.14	6.4	5.80	5.8	33	13.01	13.8	12.50	13.3
4	6.80	7	6.25	6.4	34	13.17	14	12.67	13.5
5	7.24	7.5	6.64	6.9	35	13.33	14.2	12.82	13.7
6	7.67	7.9	7.08	7.3	36	13.50	14.3	12.95	13.9
7	8.00	8.3	7.40	7.6	37	13.65	14.5	13.09	14
8	8.30	8.6	7.70	7.9	38	13.77	14.7	13.25	14.2
9	8.60	8.9	8.02	8.2	39	13.88	14.8	13.38	14.4
10	8.90	9.2	8.29	8.5	40	14.00	15	13.48	14.6
11	9.17	9.4	8.55	8.7	41	14.13	15.2	13.61	14.8
12	9.40	9.6	8.78	8.9	42	14.25	15.3	13.73	15
13	9.62	9.9	9.01	9.2	43	14.38	15.5	13.85	15.2
14	9.84	10.1	9.21	9.4	44	14.50	15.7	13.97	15.3
15	10.04	10.3	9.40	9.6	45	14.63	15.8	14.10	15.5
16	10.20	10.5	9.60	9.8	46	14.75	16	14.23	15.7
17	10.42	10.7	9.80	10	47	14.88	16.2	14.36	15.9
18	10.62	10.9	10.00	10.2	48	15.00	16.3	14.50	16.1
19	10.81	11.1	10.20	10.4	49	15.14	16.5	14.63	16.3
20	11.00	11.3	10.40	10.6	50	15.29	16.7	14.75	16.4
21	11.18	11.5	10.60	10.9	51	15.43	16.8	14.88	16.6
22	11.35	11.8	10.80	11.1	52	15.57	17	15.00	16.8
23	11.53	12	11.00	11.3	53	15.71	17.2	15.11	17
24	11.70	12.2	11.20	11.5	54	15.86	17.3	15.25	17.2
25	11.86	12.4	11.17	11.7	55	16.00	17.5	15.36	17.3
26	12.00	12.5	11.33	11.9	56	16.14	17.7	15.50	17.5
27	12.14	12.7	11.50	12.1	57	16.30	17.8	15.67	17.7
28	12.29	12.9	11.67	12.3	58	16.50	18	15.90	17.9
29	12.43	13.1	11.83	12.5	59	16.67	18.2	16.20	18

## 4. Discussion

As shown in Figures 1 & 2 the quantile regression represent more details of the growth pattern. The velocity of growth from birth to 6 months of infant age is very high and then reduced. The weight of children increased with a monotone trend after 6 months to 5 years of child age. Also it is observed that the quantile regression approach could detect and determined a suddenly weight loss at 24 months of infant age in girls this weight loss also observed for boys at 30 months of ages (Figure 1 & 2) but it is not appear in WHO Curves (Figure 4). This interesting result may be due to breastfeeding cessation at 24 and 30 month of infant ages respectively among girls and boys. Most of the mothers in Iran breastfed their babies till 24 months after birth due to following recommendation from Holy Quran “Mothers should breastfed their infants for two whole years” (Surah Baqarah verse 233, Surah Luqman verse 14, Surah Ahqaaf verse 15). But it is a traditional belief among Iranian families that the age of breastfeeding cessation for boys is after 26 months of infant age.

As shown in figures 4 the growth curves of Iranian children were similar to WHO reference curve for Infants between 0-24 months but after it the Iranian growth curves were below the WHO curves. So the use of WHO reference curves for Iranian children after 2 years were not appropriate.

## 5. Conclusion

In compare with parametric and semi-parametric methods, quantile regression (QR) as a flexible method which doesn’t require restricted assumptions and detect more details of growth pattern, proposed for estimation reference curves and normal values (Wei, Pere, & Koenker, 2006). Generally, there is a very strong agreement between the *LMS* and quantile regression growth curves estimations methods as shown in some other researches (Gannoun et al., 2002; Wei et al., 2006; Aslam & Altaf, 2011). In the present study, for all ages the agreement is extremely close (see Figure 3). The basic message is that the two approaches produce very consistent, complementary results for the estimation of growth curves.

Quantile regression is suggested as a useful method determining the reference growth curves and reference values for other age-dependent variables of medical measurements.

Some differences between growth patterns of children living in the northeast of Iran versus international ones necessitate using local and regional standards and growth curves. International norms may not properly recognize the populations at risk for growth problems among Iranian children. Therefore, they may be misleading for healthcare system.
